# Radiomic features from intratumoral and peritumoral regions on portal venous phase CT for multicenter prediction of TP53 mutation in pancreatic cancer

**DOI:** 10.3389/fonc.2026.1819664

**Published:** 2026-06-10

**Authors:** Shuyu Zhang, Xin Song, Kang Fu, Jie Liu

**Affiliations:** 1 Department of General Surgery III, Qingdao Traditional Chinese Medicine Hospital (Qingdao Hiser Hospital Affiliated of Qingdao University), Qingdao, China; 2 Department of Complaints and Appeals Office, Qingdao Traditional Chinese Medicine Hospital (Qingdao Hiser Hospital Affiliated of Qingdao University), Qingdao, China; 3 Department of Pancreatic Surgery, The Affiliated Hospital of Qingdao University, Qingdao, China; 4 Department of General Surgery,Peking University People's Hospital, Qingdao, China

**Keywords:** machine learning, pancreatic ductal adenocarcinoma, peritumoral region, radiomics, TP53

## Abstract

**Background:**

TP53 mutation, occurring in 50–70% of pancreatic ductal adenocarcinomas (PDAC), is a major determinant of tumor aggressiveness and treatment response. Current assessments rely on invasive biopsy, underscoring the need for reliable non-invasive prediction.

**Methods:**

In this multicenter study, 216 PDAC patients (training = 162; external test = 54) who underwent preoperative portal-venous phase CT (PV-phase CT) were analyzed. Intratumoral and 3-mm peritumoral regions were manually segmented, and 1, 561 radiomic features were extracted. Six machine-learning classifiers were trained following feature selection and SMOTE, both of which were strictly nested within the cross-validation training folds to prevent data leakage. Model performance was evaluated by AUC, DeLong test, decision curve, and calibration analyses; interpretability was assessed using SHAP.

**Results:**

The Intra-Peri Model (IPM) combining intratumoral and peritumoral features achieved the best performance. The XGBoost classifier yielded an AUC of 0.893 (95% CI, 0.781–1.000) in the external test set, significantly outperforming single-region models (P < 0.05). SHAP analysis identified intratumoral gray-level skewness and peritumoral texture correlation as the most influential predictors, where greater intratumoral asymmetry and lower peritumoral correlation indicated higher likelihood of TP53 mutation.

**Conclusion:**

Integrating intratumoral and peritumoral radiomics enables accurate, non-invasive prediction of TP53 status in PDAC. This model serves as a promising auxiliary tool for individualized treatment planning, warranting further prospective validation.

## Introduction

Pancreatic ductal adenocarcinoma (PDAC) is the most prevalent and aggressive subtype of pancreatic cancer, accounting for more than 90% of cases and exhibiting a dismal 5-year survival rate of approximately 6% (1–3). Its poor prognosis is largely driven by late diagnosis, rapid progression, and extensive molecular heterogeneity (4, 5). Therefore, there is an urgent and unmet clinical need for early diagnosis and non-invasive biomarkers to guide precision oncology in PDAC (6). Among the key genetic drivers, KRAS mutations occur in over 90% of cases, while TP53 mutations—present in 50–70%—represent a pivotal determinant of tumor aggressiveness and therapeutic resistance (7). The TP53-encoded p53 protein regulates cell-cycle arrest, DNA repair, and apoptosis; loss of p53 function disrupts these processes, leading to uncontrolled proliferation, genomic instability, and enhanced invasiveness (8). Accordingly, preoperative identification of TP53 mutation status has major implications for treatment selection, chemosensitivity prediction, and prognostic stratification in PDAC.

At present, TP53 mutation is typically assessed by next-generation sequencing (NGS) of surgical or biopsy tissue (9). However, this approach is invasive, carries procedural risks, and is often unsuitable for patients with advanced disease or poor performance status (10). Furthermore, the marked spatial heterogeneity of PDAC limits the representativeness of single-site biopsies, introducing sampling bias and reducing diagnostic reliability (11). These challenges highlight the urgent need for a non-invasive and comprehensive strategy to infer TP53 mutational profiles.

Radiomics offers a promising solution by converting routine medical images into high-dimensional quantitative data that reflect underlying tumor biology (12, 13). Its extension, radiogenomics, links imaging phenotypes with genomic alterations and has been successfully applied in breast, lung, and brain tumors (14–16). In PDAC, radiomics-based approaches have shown potential in predicting survival outcomes, histological subtypes, and lymph-node metastasis (17–19). Notably, Iwatate et al. (20)reported that expanding the CT region of interest (ROI) by 4 mm to include peritumoral tissue improved predictive performance (AUC = 0.795), suggesting that peritumoral characteristics may encode valuable genetic information.

The peritumoral region—representing the tumor–host interface—captures both the extent of invasion and the microenvironmental dynamics, such as stromal remodeling, inflammatory infiltration, and aberrant angiogenesis (21). Incorporating peritumoral features has enhanced model performance in predicting postoperative recurrence in PDAC and lymph-node metastasis in ovarian cancer (22, 23). Given that TP53 mutation drives epithelial–stromal crosstalk and promotes microenvironmental alterations, we hypothesize that such changes may be reflected in the peritumoral imaging phenotype. Integrating intratumoral and peritumoral radiomic features could therefore enable a more accurate, biologically grounded prediction of TP53 mutation.

Based on this premise, we conducted a multicenter study to develop and validate a machine-learning model that integrates PV-phase CT features from both intratumoral and peritumoral regions. Our goal was to establish a robust, interpretable, and non-invasive framework for preoperative TP53 mutation prediction in PDAC, thereby supporting individualized risk stratification and advancing precision oncology in pancreatic cancer.

## Materials and methods

### Study design and patient selection

This retrospective multicenter study was approved by the Ethics Committees of the Affiliated Hospital of Qingdao University and Peking University People’s Hospital (approval numbers: QYFY-WZLL-30714, 2025PHQDB030-01). Given its retrospective design, the requirement for informed consent was waived. All patient data were fully de-identified prior to analysis to ensure confidentiality. All methods were performed in accordance with the Declaration of Helsinki. TP53 mutational data were obtained from next-generation sequencing (NGS) conducted by BGI Genomics.

Inclusion criteria: (1) pathologically confirmed pancreatic ductal adenocarcinoma (PDAC) between January 2019 and June 2025 at the two participating centers; (2) availability of contrast-enhanced upper abdominal CT within one month before surgery; (3) confirmed TP53 mutational status by NGS of tumor tissue; and (4) complete imaging data without significant artifacts.

**Figure 1:**
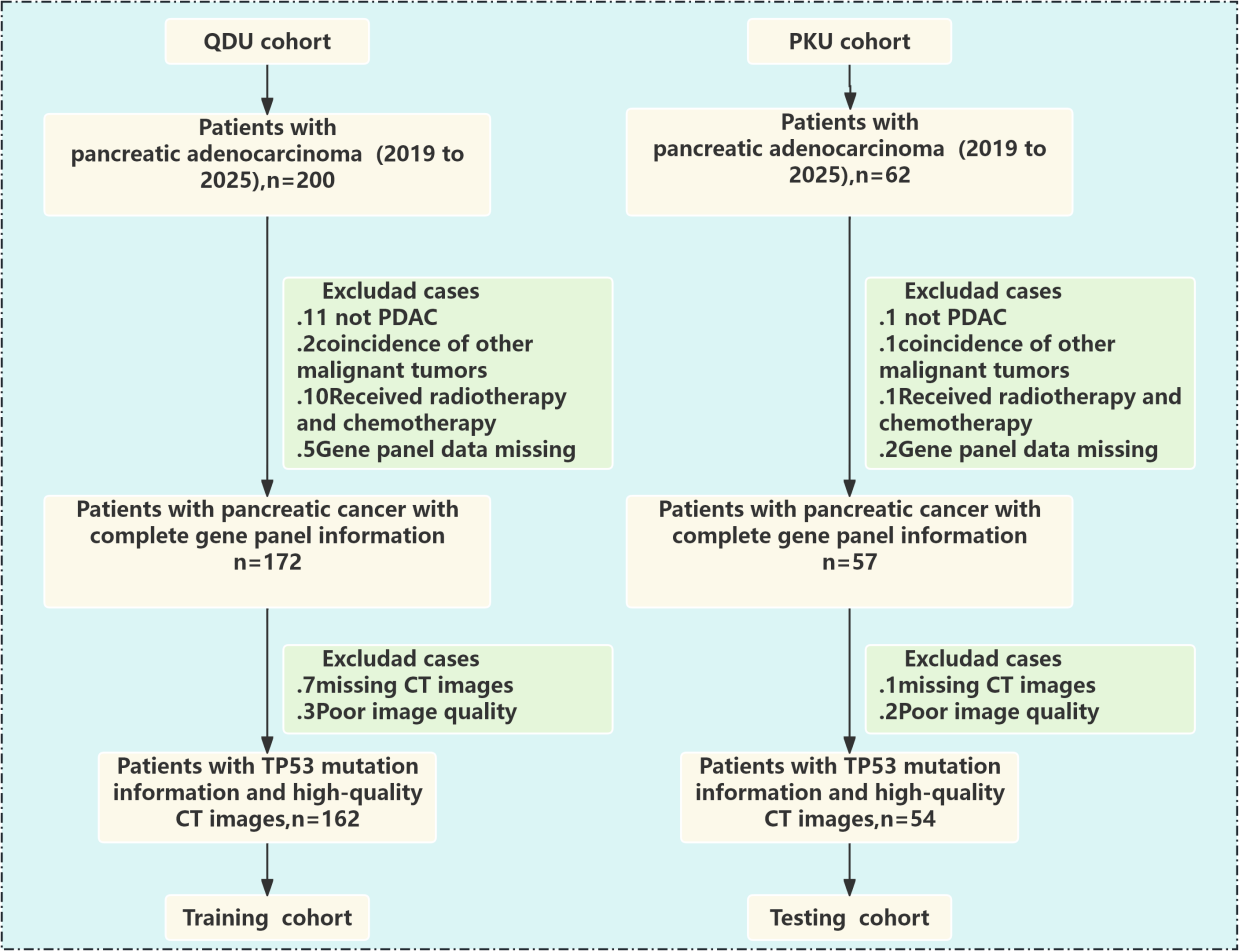
Flowchart of patient enrollment and exclusion criteria for QDU and PKU in TP53 mutation prediction study of pancreatic adenocarcinoma.

Exclusion criteria: (1) prior neoadjuvant chemotherapy, radiotherapy, targeted therapy, or immunotherapy before CT; (2) severe motion or metal artifacts affecting feature extraction; (3) poorly defined tumor boundaries; (4) concurrent malignancies; or (5) missing TP53 mutation data (**Figure 1**).

The following variables were extracted from electronic medical records: Baseline demographics(Age, gender, BMI);Comorbidities(DM, Pancreatitis, Hyperlipidemia);Laboratory parameters(platelet[PLT], lymphocytes[Lym], neutrophils[N], hemoglobin [HB], albumin[Alb], prealbumin[PAB], albumin/globulin[A/G], direct bilirubin [DBIL], indirect bilirubin[IBIL], alanine transaminase[ALT], aspartate aminotransferase [AST], fasting plasma glucose [FPG], CA199, CEA); Pathological data(Maximum tumor diameter, Ki67, T N M stage).

**Figure 2:**
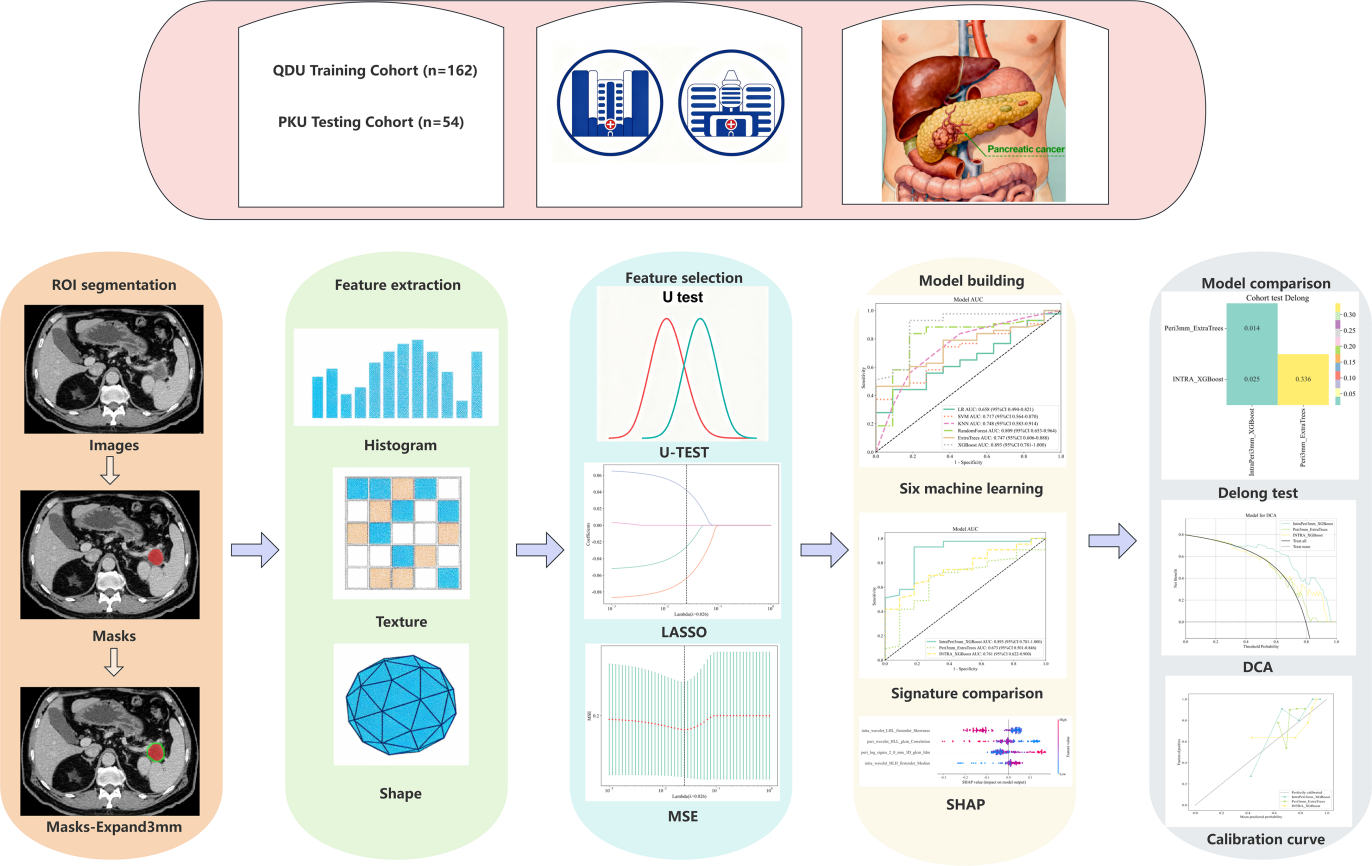
Workflow of this study.

A total of 216 patients met the inclusion criteria, including 162 cases from the Affiliated Hospital of Qingdao University (training cohort) and 54 from the Peking University People’s Hospital (external test cohort). TP53 mutations were classified as mutant-type if they were pathogenic or likely pathogenic variants, including missense, nonsense, or frameshift mutations, as defined according to the ACMG/AMP guidelines (24). Wild-type TP53 referred to samples without such pathogenic variants, including those harboring benign or likely benign variants or no detectable mutation (**Figure 2**).

### Image acquisition and preprocessing

All patients underwent three-phase contrast-enhanced upper abdominal CT using either a 64- or 128-slice spiral scanner (GE Discovery 750HD or Siemens Somatom Definition Flash). Portal venous phase images were acquired at a 70-second delay. Scanning parameters were as follows: tube voltage 120 kV, automatic tube current modulation, slice thickness 2.5 mm, and interslice gap 2.5 mm.

Image preprocessing was performed as follows: (1) window normalization to a pancreatic-specific setting (window width 350 HU, window level 40 HU); (2) gray-level normalization to a range of 0–255; (3) voxel resampling using trilinear interpolation to an isotropic resolution of 1.0 × 1.0 × 1.0 mm³ to minimize scanner-dependent variation; and (4) artifact removal by manually excluding irrelevant structures such as bowel gas and bone.

To address scanner-related variability across centers, all CT images were further normalized using z-score harmonization before feature extraction. This step minimized inter-scanner intensity and texture distribution differences and ensured feature comparability across the multicenter dataset.

### ROI segmentation and reproducibility assessment

Tumor segmentation was performed manually on portal venous phase CT images using ITK-SNAP software (v3.8.0). Two radiologists (each with >5 years of experience in abdominal imaging) delineated the intratumoral regions of interest (ROIs) slice by slice, including only viable tumor tissue and excluding adjacent normal pancreas, vessels, or surrounding organs. Discrepancies were reviewed by a senior radiologist with >10 years of experience to generate a consensus ROI.

Peritumoral ROIs were obtained by isotropically expanding the consensus tumor contour by 3 mm to form an annular region. To determine the optimal peritumoral region, we compared the performance of features extracted from 2-mm and 3-mm margins within the training cohort. The 3-mm margin demonstrated superior stability and predictive accuracy during cross-validation (**Supplementary Figures 1**, **S2**). Therefore, the 3-mm margin was selected for final model construction. Subsequently, any portions extending into air cavities, bone, or non-soft-tissue structures were manually excluded to ensure accurate representation of the peritumoral microenvironment.

To evaluate interobserver consistency, 50 cases were randomly selected for repeated segmentation by a second radiologist. The intraclass correlation coefficient (ICC) was calculated for all extracted radiomic features, with ICC ≥ 0.75 indicating good agreement.

### Radiomic feature extraction

Radiomic feature extraction was performed using Python 3.9 and the PyRadiomics package (v3.0.1). The workflow included: (1) image transformation with Gaussian Laplacian filtering (sigma = 1.0, 2.0, 3.0) and wavelet decomposition, generating 11 derived image sets; (2) feature computation across seven categories—shape, first-order statistics, gray-level co-occurrence matrix (GLCM), gray-level run-length matrix (GLRLM), gray-level size zone matrix (GLSZM), gray-level dependence matrix (GLDM), and neighborhood gray-tone difference matrix (NGTDM); and (3) total feature count—1, 561 features extracted separately from intratumoral and peritumoral ROIs (**Supplementary Table 1**).

### Feature selection

To reduce dimensionality and redundancy, a four-step selection pipeline was applied. All feature selection steps (U test, correlation, mRMR, and LASSO) were strictly nested within the training folds of the cross-validation process:

1. Univariate filtering: Mann–Whitney U test identified features significantly associated with TP53 mutation status (P < 0.05).
2. Correlation analysis: Highly correlated features (|r| > 0.90) were removed to reduce multicollinearity.
3. mRMR filtering: The minimum redundancy–maximum relevance algorithm selected the top features most relevant to TP53 mutation while minimizing redundancy.
4. LASSO regression: Five-fold cross-validation determined the optimal regularization parameter (λ) based on the minimum binomial deviance (λ_min). Features with nonzero coefficients were retained for model construction.
5. For the Intra-Peri Model, intratumoral and peritumoral features were concatenated into a single dataset before applying the same four-step pipeline to identify the optimal fusion signature.

### Model construction and hyperparameter optimization

Three models were developed based on the selected features: (1) intratumoral-only, (2) peritumoral-only, and (3) intra-peri model. Six machine-learning classifiers were tested—logistic regression (LR), support vector machine (SVM), k-nearest neighbor (KNN), random forest (RF), extremely randomized trees (ExtraTrees), and extreme gradient boosting (XGBoost).

Model training used five-fold cross-validation within the training cohort: the dataset was randomly split into five folds, with four folds used for training and one for validation in each iteration. To mitigate the class imbalance caused by the higher TP53 mutation rate in the training cohort (73.46% vs. 26.54%), data augmentation was applied during model training. Specifically, minority-class samples (wild-type TP53) were synthetically oversampled using the Synthetic Minority Over-sampling Technique (SMOTE) exclusively within the training folds of the cross-validation loop. The original class distribution was preserved in all validation folds and the external test cohort to reflect real-world clinical prevalence and avoid biased performance evaluation. Additionally, class weights were adjusted in algorithms that support weighted learning. The median AUC across folds represented training performance, reducing bias from random sampling. Key hyperparameters were tuned using grid search based on cross-validated AUC. Detailed parameter ranges are provided in **Supplementary Table 2**.

### Model evaluation

Model performance was primarily assessed in the external test cohort using the area under the receiver operating characteristic curve (AUC), accuracy, sensitivity, specificity, positive predictive value (PPV), negative predictive value (NPV), and F1 score.

Comparisons between models were performed using the DeLong test (P < 0.05 considered statistically significant). Decision curve analysis (DCA) evaluated clinical net benefit across threshold probabilities relative to “treat all” and “treat none” strategies. Calibration curves were used to assess agreement between predicted and observed probabilities; closer alignment with the 45° reference line indicated better calibration.

### SHAP explainability analysis

The SHAP (SHapley Additive exPlanations) framework was applied to the best-performing model to interpret feature contributions. SHAP values were computed for all features to quantify importance and visualize feature–outcome relationships using summary plots. Individual-level explanations were also generated to illustrate prediction logic and model transparency.

### Statistical analysis

All analyses were conducted using Python 3.9 (Scikit-learn, PyRadiomics, and SHAP packages) and SPSS version 26.0 (IBM Corp.). Continuous variables were expressed as mean ± standard deviation (SD) and compared using the Mann-Whitney U-test; categorical variables were presented as counts (percentages) and compared using the χ² test. A two-sided P < 0.05 was considered statistically significant.

## Results

### Clinical baseline characteristics

The clinical characteristics of patients in the training and external test cohorts are summarized in **Table 1**. No significant differences were observed between cohorts in TP53 mutation status, sex, age, BMI, history of diabetes or pancreatitis, maximum tumor diameter, TNM stage or serum tumor markers (CA19–9 and CEA) (all P > 0.05), except for AST levels, which showed a mild but clinically insignificant difference (P = 0.033), indicating good overall comparability for model development and validation. The TP53 mutation rate was 73.46% (119/162) in the training cohort and 79.63% (43/54) in the test cohort.

**Table 1:** Baseline clinical and demographic characteristics of patients in the training and test cohorts.

Feature_name	ALL(n = 216)	Test(n = 54)	Train(n = 162)	P value
TP53, n(%)				0.468
No	54(25.00)	11(20.37)	43(26.54)	
Yes	162(75.00)	43(79.63)	119(73.46)	
Sex, n(%)				1.0
Male	128(59.26)	32(59.26)	96(59.26)	
Female	88(40.74)	22(40.74)	66(40.74)	
DM, n(%)				0.346
No	151(69.91)	41(75.93)	110(67.90)	
Yes	65(30.09)	13(24.07)	52(32.10)	
Pancreatitis, n(%)				0.62
No	203(93.98)	52(96.30)	151(93.21)	
Yes	13(6.02)	2(3.70)	11(6.79)	
Hyperlipidemia, n(%)				0.465
No	81(37.50)	23(42.59)	58(35.80)	
Yes	135(62.50)	31(57.41)	104(64.20)	
T stage, n(%)				0.417
1	13(6.02)	5(9.26)	8(4.94)	
2	98(45.37)	20(37.04)	78(48.15)	
3	64(29.63)	17(31.48)	47(29.01)	
4	41(18.98)	12(22.22)	29(17.90)	
N stage, n(%)				0.479
0	107(49.54)	23(42.59)	84(51.85)	
1	97(44.91)	28(51.85)	69(42.59)	
2	12(5.56)	3(5.56)	9(5.56)	
M stage, n(%)				0.176
0	161(74.54)	36(66.67)	125(77.16)	
1	55(25.46)	18(33.33)	37(22.84)	
Age (years), Mean ± SD	62.96 ± 9.43	61.30 ± 10.46	63.51 ± 9.03	0.204
BMI(kg/m2), Mean ± SD	23.40 ± 3.52	23.39 ± 3.28	23.41 ± 3.60	0.999
PLT(10^9/L), Mean ± SD	227.29 ± 78.23	227.09 ± 85.54	227.35 ± 75.92	0.974
N (10^9/L), Mean ± SD	4.83 ± 4.26	4.99 ± 3.70	4.77 ± 4.44	0.901
Lym(10^9/L), Mean ± SD	1.74 ± 2.83	1.66 ± 1.08	1.77 ± 3.21	0.324
Alb(g/L), Mean ± SD	41.11 ± 13.89	39.57 ± 4.57	41.62 ± 15.80	0.248
PAB(g/L), Mean ± SD	217.10 ± 67.73	207.96 ± 67.40	220.15 ± 67.78	0.253
A/G, Mean ± SD	2.74 ± 15.59	1.63 ± 0.28	3.11 ± 18.00	0.199
DBIL(umol/L), Mean ± SD	48.91 ± 79.68	53.87 ± 74.39	47.26 ± 81.52	0.28
IBIL(umol/L), Mean ± SD	18.12 ± 22.14	19.43 ± 21.19	17.69 ± 22.49	0.674
ALT(U/L), Mean ± SD	102.85 ± 165.09	102.87 ± 134.76	102.84 ± 174.41	0.291
AST(U/L), Mean ± SD	63.45 ± 85.18	73.98 ± 96.04	59.94 ± 81.25	0.033
FPG (mmol/L), Mean ± SD	7.23 ± 2.78	6.73 ± 2.41	7.39 ± 2.89	0.157
CA199(U/mL), Mean ± SD	3269.34 ± 19530.62	9240.49 ± 37855.63	1278.96 ± 4657.18	0.552
CEA(ng/mL), Mean ± SD	24.98 ± 138.53	18.71 ± 43.44	27.07 ± 158.08	0.391
Maximum tumor diameter (cm), Mean ± SD	3.91 ± 1.59	3.99 ± 1.38	3.88 ± 1.66	0.206
Ki67(%), Mean ± SD	31.39 ± 13.20	31.02 ± 13.89	31.52 ± 13.00	0.762

SD, standard deviation.

### Feature extraction

**Figure 3:**
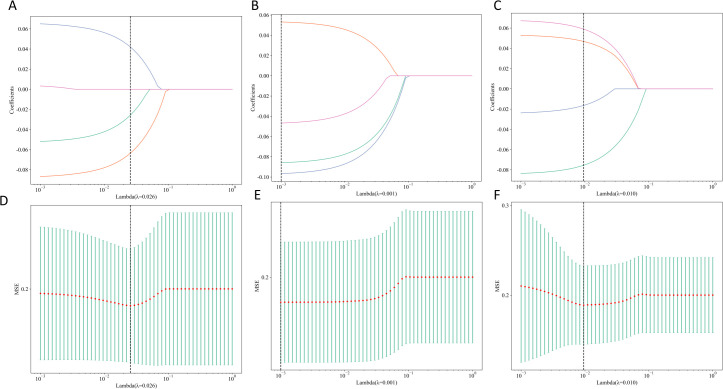
Visualization of extracted radiomics features: **(A)** Proportions and Counts of Feature Classes, **(B)** Violin Plots of Feature Value Distributions.

A total of 1, 561 radiomic features were extracted from each region of interest using the PyRadiomics package (**Figure 3**).

### Feature selection

**Figure 4:**
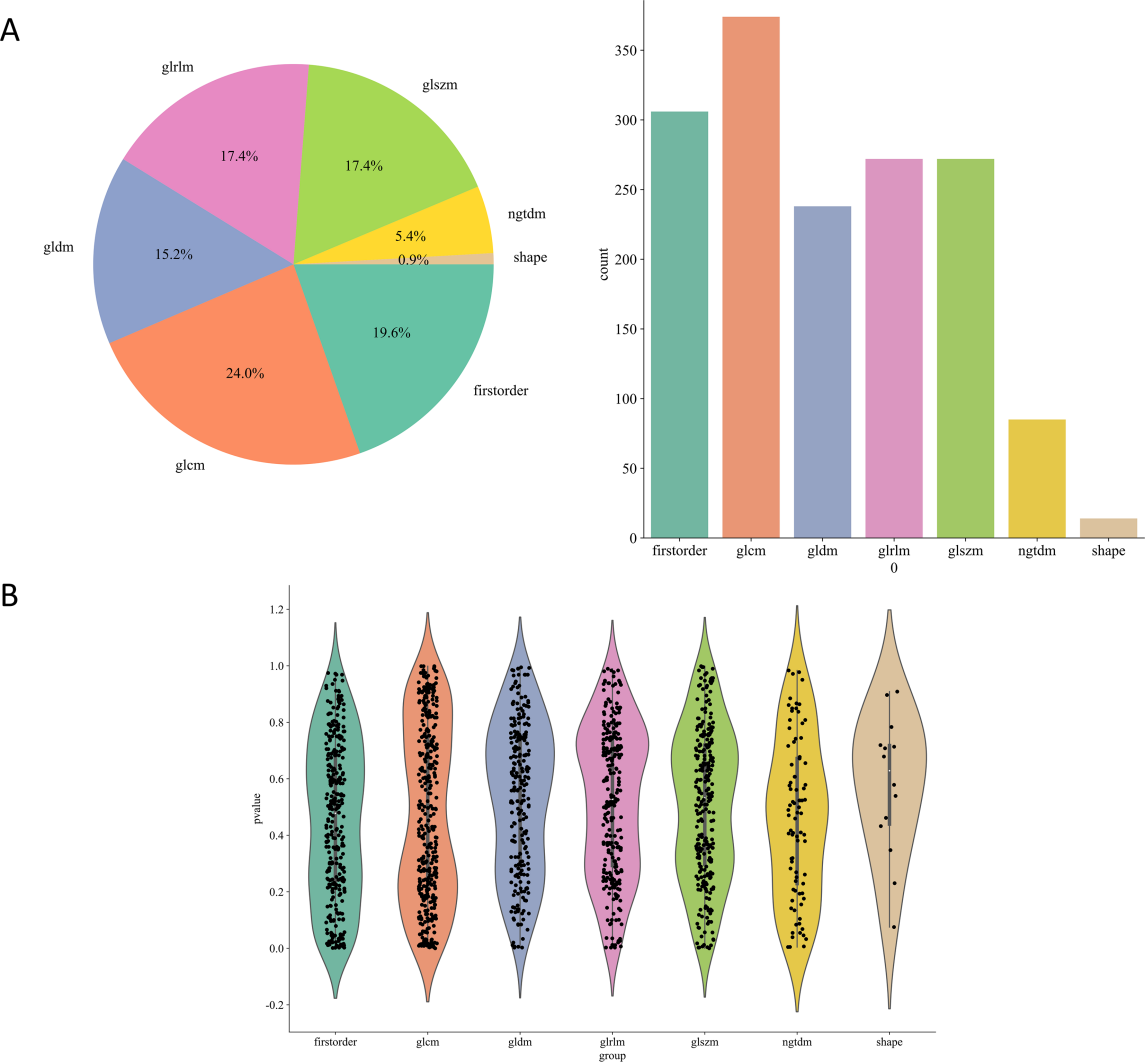
LASSO regularization of radiomics features: **(A–C)** Coefficient Paths and **(D–F)** MSE Profiles for Intratumoral, Peritumoral, and Intra-Peri Feature Sets.

**Figure 5:**
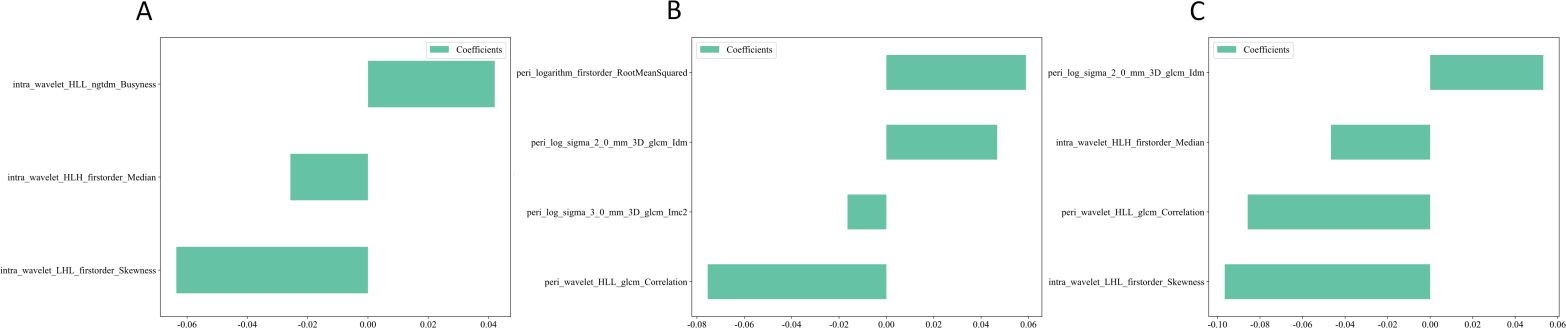
Coefficient bar plots of final selected radiomics features for **(A)** Intratumoral, **(B)** Peritumoral, and **(C)** Intra-Peri.

After multistep selection (**Figure 4**), the final feature sets were as follows:

Intratumoral model: three features — wavelet-HLL_ngtdm_Busyness, wavelet-HLH_firstorder_Median, and wavelet-LHL_firstorder_Skewness (**Figure 5A**).

Peritumoral model: four features — logarithm_firstorder_RootMSquare, log-sigma-2-0-mm-3D_glcm_LDH, log-sigma-3-0-mm-3D_glcm_LMC, and wavelet-HLL_glcm_Correlation(**Figure 5B**).

Intra-Peri Model: four features, including two intratumoral (wavelet-HLH_firstorder_Median, wavelet-LHL_firstorder_Skewness) and two peritumoral (log-sigma-2-0-mm-3D_glcm_LDH, wavelet-HLL_glcm_Correlation) variables (**Figure 5C**).

### Model performance comparison

#### AUC comparison among models

For the intratumoral model, XGBoost achieved the best performance (AUC = 0.761). The peritumoral model yielded lower overall performance, with ExtraTrees showing the highest AUC (0.673; range 0.542–0.653 for other algorithms).

**Figure 6:**
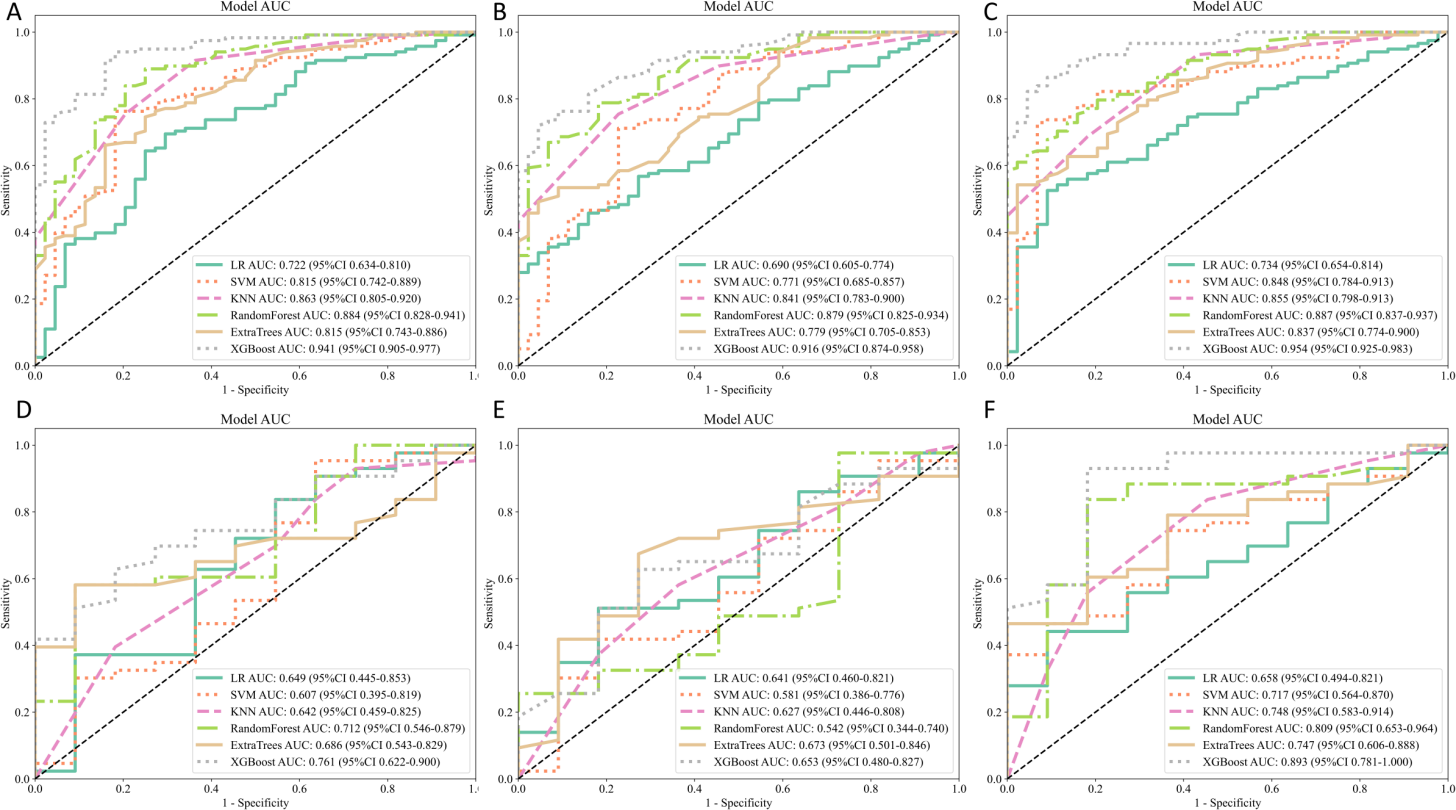
ROC curves of six machine learning algorithms for TP53 mutation prediction in pancreatic cancer: **(A–C)** Training Set (Intratumoral, Peritumoral, Intra-Peri) and **(D–F)** Testing Set (Intratumoral, Peritumoral, Intra-Peri) with AUC and 95% Confidence Intervals.

**Figure 7:**
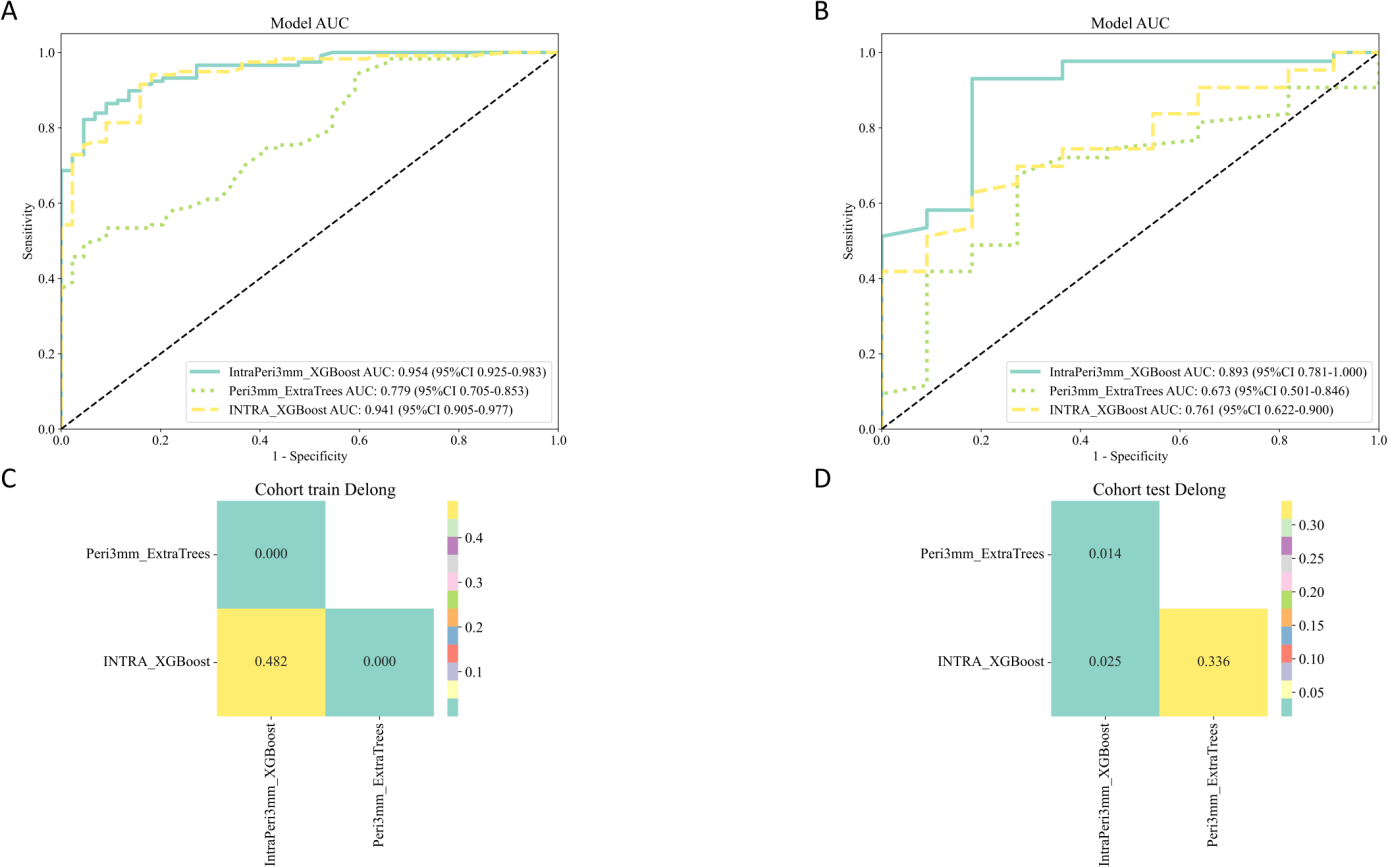
Comparative analysis of best-performing machine learning models for TP53 mutation prediction in pancreatic cancer: **(A, C)** Training Set and **(B, D)** Testing Set with ROC Curves and Delong Test for Intratumoral, Peritumoral, and Intra-Peri Models.

The intra-peri model consistently outperformed the other two across all algorithms. The XGBoost-based intra-peri model achieved the best discrimination, with an AUC of 0.893 (95% CI, 0.781–1.000) (**Figure 6**), significantly higher than the intratumoral XGBoost model (AUC = 0.761, P = 0.025) and the best peritumoral model (ExtraTrees, AUC = 0.673, P = 0.014). (**Figure 7**; **Table 2**).

**Table 2:** Predictive performance of the intratumoral, peritumoral, and intra-peri models for TP53 mutation status in the training and test cohorts.

model_name	Accuracy	AUC	95% CI	Sensitivity	Specificity	Cohort
Peritumoral model						
LR	0.562	0.690	0.6050 - 0.7741	0.458	0.841	train
LR	0.574	0.641	0.4603 - 0.8208	0.512	0.818	test
SVM	0.728	0.771	0.6846 - 0.8566	0.712	0.773	train
SVM	0.500	0.581	0.3864 - 0.7764	0.419	0.818	test
KNN	0.759	0.841	0.7828 - 0.9002	0.754	0.773	train
KNN	0.593	0.627	0.4462 - 0.8075	0.581	0.636	test
RandomForest	0.796	0.879	0.8248 - 0.9337	0.788	0.818	train
RandomForest	0.407	0.542	0.3444 - 0.7402	0.256	1.000	test
ExtraTrees	0.617	0.779	0.7047 - 0.8531	0.492	0.955	train
ExtraTrees	0.685	0.673	0.5006 - 0.8461	0.674	0.727	test
XGBoost	0.784	0.916	0.8740 - 0.9579	0.720	0.955	train
XGBoost	0.648	0.653	0.4795 - 0.8270	0.628	0.727	test
Intratumoral model						
LR	0.698	0.722	0.6336- 0.8101	0.695	0.705	train
LR	0.759	0.649	0.4451- 0.8530	0.837	0.455	test
SVM	0.778	0.815	0.7423- 0.8886	0.763	0.818	train
SVM	0.833	0.607	0.3945- 0.8190	0.953	0.364	test
KNN	0.840	0.863	0.8054- 0.9202	0.915	0.636	train
KNN	0.481	0.642	0.4585- 0.8248	0.395	0.818	test
RandomForest	0.852	0.884	0.8277- 0.9408	0.890	0.750	train
RandomForest	0.648	0.712	0.5456- 0.8794	0.581	0.909	test
ExtraTrees	0.710	0.815	0.7432- 0.8864	0.661	0.841	train
ExtraTrees	0.648	0.686	0.5426- 0.8295	0.581	0.909	test
XGBoost	0.907	0.941	0.9054- 0.9773	0.941	0.818	train
XGBoost	0.667	0.761	0.6220- 0.9002	0.628	0.818	test
Intra-Peri model						
LR	0.630	0.734	0.6544 - 0.8144	0.525	0.909	train
LR	0.537	0.658	0.4941 - 0.8209	0.442	0.909	test
SVM	0.784	0.848	0.7838 - 0.9130	0.729	0.932	train
SVM	0.722	0.717	0.5636 - 0.8698	0.744	0.636	test
KNN	0.722	0.855	0.7978 - 0.9128	0.686	0.818	train
KNN	0.778	0.748	0.5833 - 0.9136	0.837	0.545	test
RandomForest	0.778	0.887	0.8372 - 0.9367	0.754	0.841	train
RandomForest	0.833	0.809	0.6532 - 0.9641	0.837	0.818	test
ExtraTrees	0.660	0.837	0.7742 - 0.9003	0.542	0.977	train
ExtraTrees	0.574	0.747	0.6064 - 0.8883	0.465	1.000	test
XGBoost	0.858	0.954	0.9255 - 0.9830	0.822	0.955	train
XGBoost	0.907	0.893	0.7814 - 1.0000	0.930	0.818	test

#### Decision curve and calibration analyses

**Figure 8:**
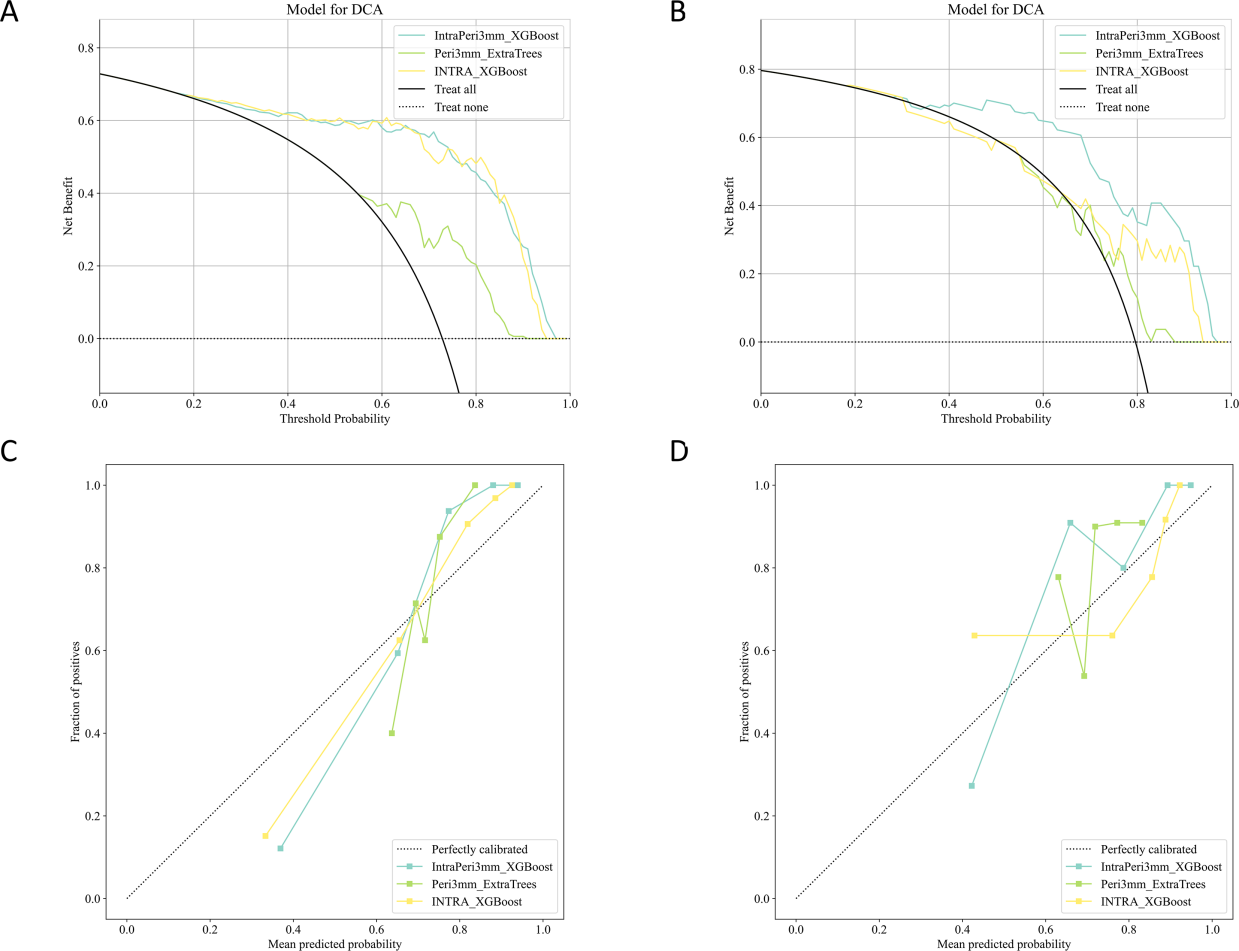
Decision curve analysis (DCA) and calibration curves for TP53 mutation prediction models: **(A, C)** Training Set and **(B, D)** Testing Set.

Decision curve analysis (DCA) demonstrated that the intra-peri XGBoost model provided greater net clinical benefit across a threshold probability range of 0.1–0.8 compared with the intratumoral XGBoost, peritumoral ExtraTrees, and “treat-all” or “treat-none” strategies, confirming superior clinical utility. (**Figure 8B**).

Calibration analysis revealed strong agreement between predicted and observed probabilities for the intra-peri XGBoost model, with the curve closely aligned to the 45° reference line. The intratumoral model showed mild deviation, whereas the peritumoral model displayed notable bias, indicating optimal calibration for the fused model. (**Figure 8D**).

#### SHAP-based model interpretability

**Figure 9:**
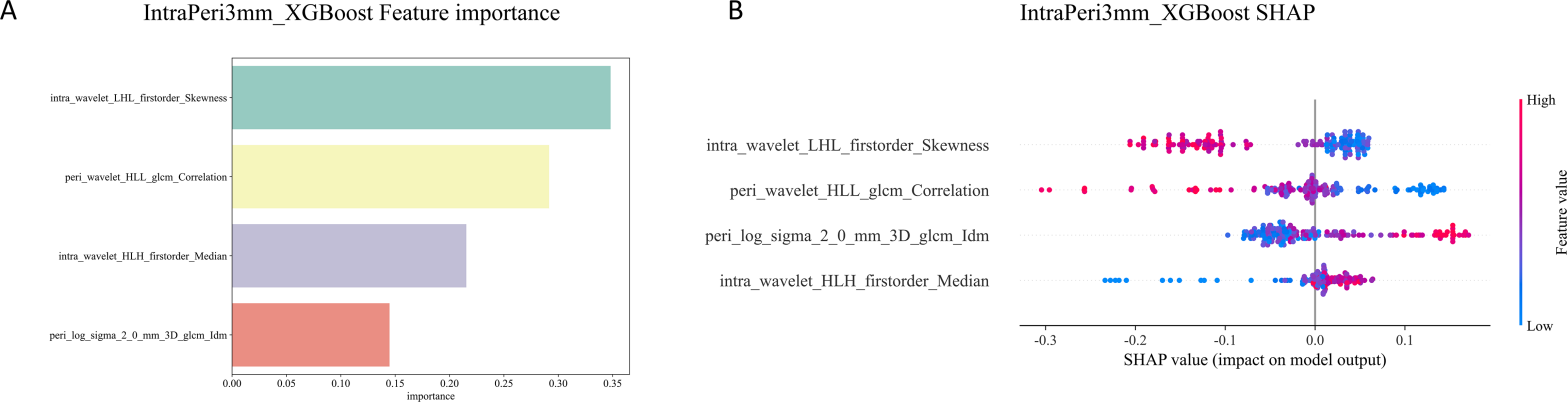
Feature interpretation of intra-peri XGBoost model for TP53 mutation Prediction: **(A)** Feature importance and **(B)** SHAP analysis.

SHAP analysis of the intra-peri XGBoost model identified intratumoral wavelet-LHL_firstorder_Skewness (gray-level skewness) and peritumoral wavelet-HLL_glcm_Correlation (texture correlation) as the two most influential predictors with the highest SHAP contributions. (**Figure 9A**).

Higher intratumoral skewness—reflecting increased asymmetry and textural irregularity—and lower peritumoral correlation—indicating greater tissue heterogeneity—were associated with a higher predicted probability of TP53 mutation, whereas the opposite patterns favored wild-type classification. (**Figure 9B**).

**Figure 10:**
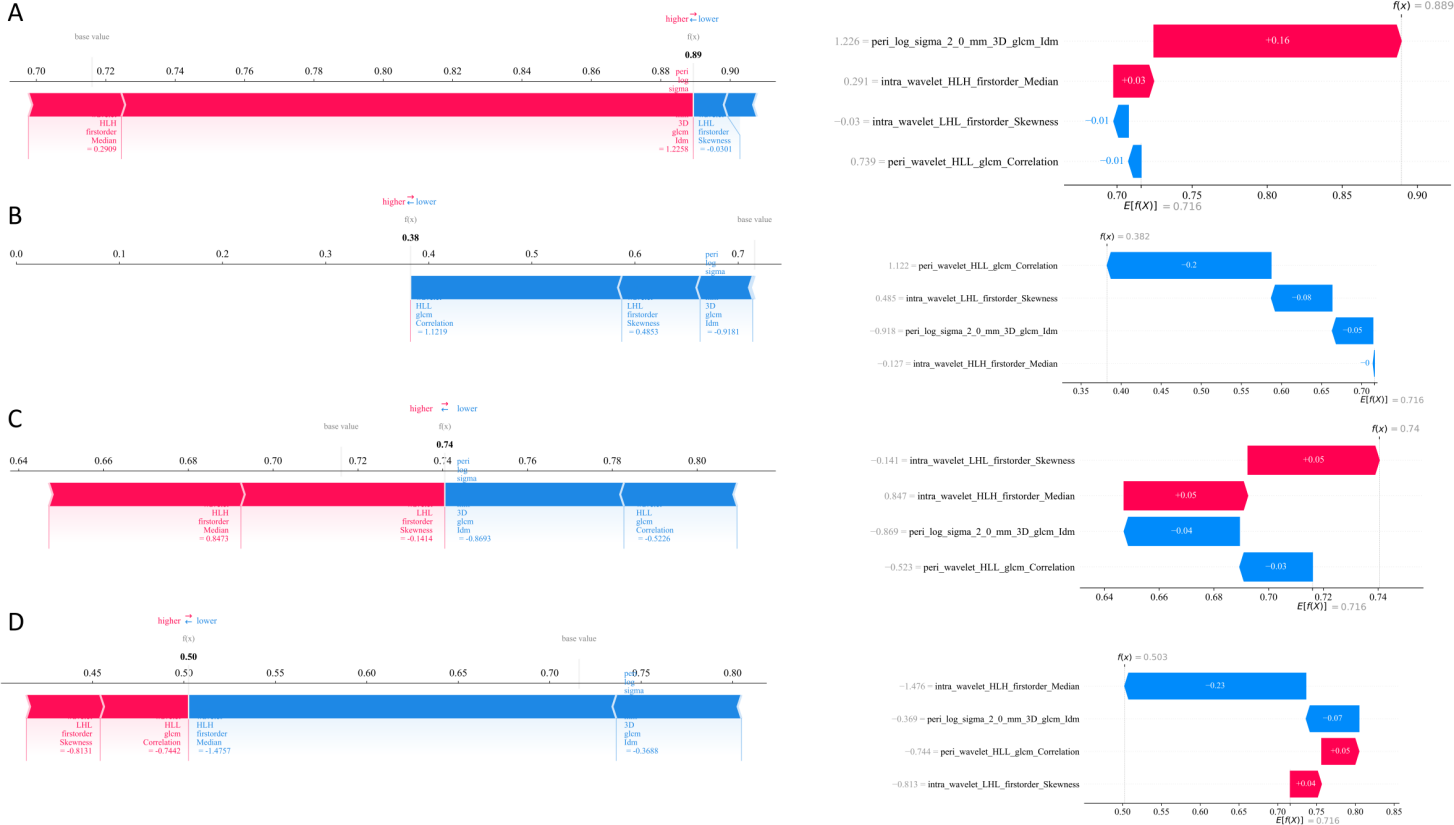
Individual SHAP analysis of Intra-Peri XGBoost model for TP53 mutation prediction: **(A)** True Positive, **(B)** True Negative, **(C)** False Positive, and **(D)** False Negative Cases.

At the individual-case level, true-positive and true-negative samples exhibited consistent feature-contribution trends, while false-positive and false-negative cases showed conflicting local feature effects (e.g., high intratumoral skewness in wild-type lesions), suggesting that misclassification primarily arose from boundary cases with complex textural heterogeneity. (**Figure 10**).

## Discussion

Pancreatic ductal adenocarcinoma (PDAC) is an aggressive malignancy characterized by marked molecular and radiological heterogeneity. Mutations in driver genes play pivotal roles in tumor progression, among which TP53 alterations are closely associated with enhanced invasiveness, therapeutic resistance, and poor prognosis, and may also modulate tumor–microenvironment interactions (25, 26). Conventional molecular testing relies on tissue biopsy, which is limited by sampling bias and spatial heterogeneity, making dynamic or preoperative assessment challenging (27). Radiomics provides a noninvasive approach to quantitatively characterize tumor phenotypes from standard medical images, offering the potential to infer underlying molecular profiles (28). If TP53 status can be reliably predicted from PV-phase CT—the most commonly used imaging modality in PDAC management—it could substantially improve risk stratification, guide treatment planning, and facilitate clinical trial enrollment.

In this multicenter study, we developed and validated a radiomics model integrating intratumoral and peritumoral features for noninvasive prediction of TP53 mutation status in PDAC. The intra-peri XGBoost model achieved an AUC of 0.893 in the independent external cohort, significantly outperforming models based on single-region features. The model demonstrated clear biological interpretability: SHAP analysis identified intratumoral wavelet-LHL_firstorder_Skewness (gray-level skewness) and peritumoral wavelet-HLL_glcm_Correlation (texture correlation) as the most influential features. Elevated intratumoral skewness reflected increased compositional complexity and architectural irregularity within the tumor, whereas reduced peritumoral correlation indicated disruption of stromal organization and enhanced heterogeneity in surrounding tissues (29, 30). Together, these findings delineate an imaging phenotype associated with TP53 inactivation—characterized by internal disorganization (“skewed” intensity distributions) and peripheral textural “decorrelation.” The former likely arises from TP53-driven genomic instability, metabolic stress, and necrotic–fibrotic interplay within the tumor (31), while the latter reflects invasive behavior and epithelial–mesenchymal interactions that induce stromal remodeling and inflammatory infiltration at the tumor boundary (32). Thus, this “intratumoral skewness–peritumoral decorrelation” signature can be viewed as an imaging manifestation of TP53-related molecular and microenvironmental remodeling. Although SHAP provides algorithmic transparency, the exact biological basis of these radiomic features remains speculative, warranting future spatially matched radiologic-pathologic validation.

Compared with previous studies, our work provides stronger evidence and greater clinical feasibility. Iwatate et al. reported that including a 4-mm peritumoral margin improved CT-based TP53 prediction (AUC from 0.705 to 0.795) (20). In contrast, our model achieved higher discrimination (AUC = 0.893) using only portal venous phase CT by optimizing feature selection and algorithmic strategy, and was externally validated across multiple centers, demonstrating enhanced robustness and generalizability. Gao et al. achieved an AUC of 0.96 using multiparametric MRI (33), but their approach required complex imaging protocols and lacked external validation. Our model, despite using simpler single-phase CT, achieved comparable performance, suggesting stronger translational potential. Methodologically, systematic comparison of multiple algorithms identified XGBoost as optimal for handling the high-dimensional, nonlinear, and intercorrelated radiomic feature space. The integration of SHAP interpretability further bridged model transparency and biological plausibility, forming a closed loop from data acquisition to clinical application.

Importantly, this radiomics model is intended as an auxiliary decision-support tool rather than a replacement for standard genomic testing. Clinically, preoperative prediction of TP53 status can tangibly alter management decisions. Recent evidence demonstrates that PDAC patients with wild-type TP53 exhibit significantly higher objective response rates and longer overall survival when receiving the FOLFIRINOX regimen compared to those with TP53 mutations (34). Thus, our non-invasive intra-peri model could practically guide neoadjuvant chemotherapy choices, potentially sparing TP53-mutated patients from severe toxicities in favor of alternative regimens or clinical trials. Furthermore, while we initially explored a clinico-radiomic fusion model, conventional clinical markers (e.g., CA19-9, TNM stage) did not yield significant incremental predictive value over our robust intra-peri radiomics signature alone.

Several limitations should be acknowledged. First, the retrospective design and strict requirement for both high-quality CT and NGS data restricted the sample size and introduced selection bias, precluding patients unsuitable for surgery. Second, this study relied solely on single-phase portal venous CT; the complementary value of multiphase CT or MRI remains unexplored. Third, manual ROI segmentation is operator-dependent and time-consuming. Furthermore, our empirically selected 3-mm peritumoral margin may not perfectly capture the microenvironmental heterogeneity across all tumors; future studies should integrate deep learning-based automatic segmentation (such as the state-of-the-art nnU-Net framework) and anatomically adaptive margins. Additionally, exploring advanced feature-domain harmonization methods like ComBat should be investigated to further mitigate scanner-related batch effects. Fourth, this proof-of-concept study lacks longitudinal survival endpoints; prospective randomized controlled trials are required to confirm its true clinical benefit. Finally, we did not account for co-mutations (e.g., KRAS, CDKN2A, SMAD4) that may interactively influence radiomic phenotypes, which warrants exploration in future multi-gene studies.

In summary, the multicenter-validated intra-peri radiomics model based on routine PV-phase CT enables accurate and interpretable prediction of TP53 mutation status in PDAC. By non-invasively capturing mutation-driven structural heterogeneity and stromal remodeling, it constitutes a biologically grounded, auxiliary framework for individualized risk stratification and precision therapy in pancreatic cancer.

## Conclusion

This multicenter study validated the effectiveness of a machine learning–based radiomics model that integrates intratumoral and peritumoral features from PV-phase CT for predicting TP53 mutation status in patients with pancreatic ductal adenocarcinoma (PDAC). The intra-peri XGBoost model achieved an AUC of 0.893 in the independent external cohort, significantly outperforming models built on single-region features. SHAP analysis quantified and clarified the contribution and directionality of key features, enhancing the model’s biological interpretability and clinical transparency. By enabling preoperative, noninvasive inference of TP53 status, the model offers potential decision support for individualized risk stratification, treatment planning, and precision management of PDAC. Future large-scale prospective studies are warranted to further validate the model’s robustness and clinical utility, ultimately facilitating the translation of radiogenomics into precision oncology practice for pancreatic cancer.

## Data Availability

The original contributions presented in the study are included in the article/**Supplementary Material**. Further inquiries can be directed to the corresponding author.

## Supplementary material

Supplementary Figure 1 Comparison of model performance using 2-mm and 3-mm peritumoral margins in the training cohort.

Supplementary Figure 2 Schematic illustration of the region of interest (ROI) segmentation and peritumoral margin dilation.

Supplementary Table 1 Details of the radiomics features extracted from the CT images.

Supplementary Table 2 Hyperparameter settings for the machine learning algorithms.
