# Supplementary figures and images for: Radiomic features from intratumoral and peritumoral regions on portal venous phase CT for multicenter prediction of TP53 mutation in pancreatic cancer
Source: Front Oncol. 2026 Jun 10;16:1819664. doi: 10.3389/fonc.2026.1819664 (PMC13290449; doi:10.3389/fonc.2026.1819664)

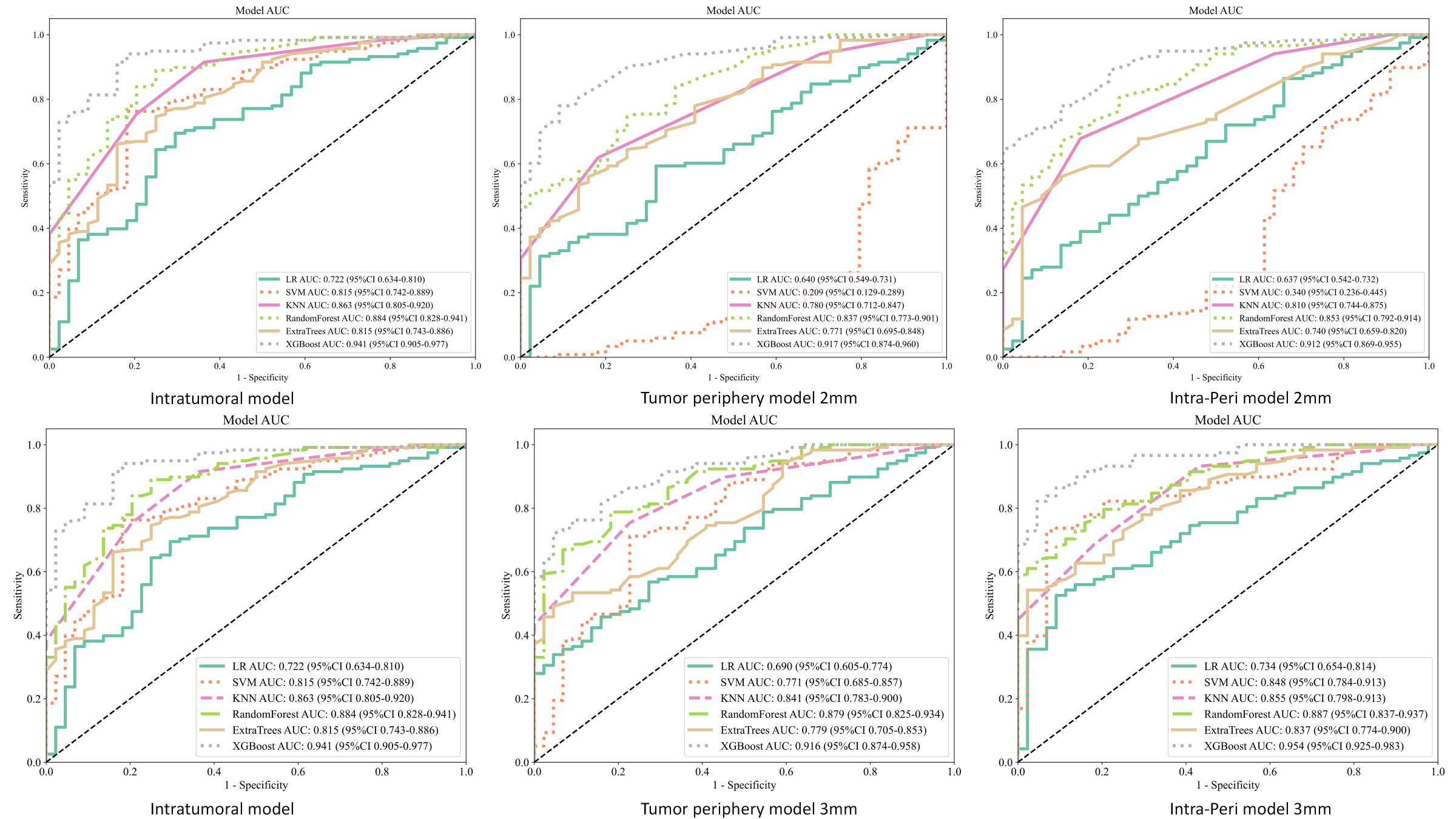

Supplement: Supplementary Figure 1 — Comparison of model performance using 2-mm and 3-mm peritumoral margins in the training cohort. [file Image1.tiff]

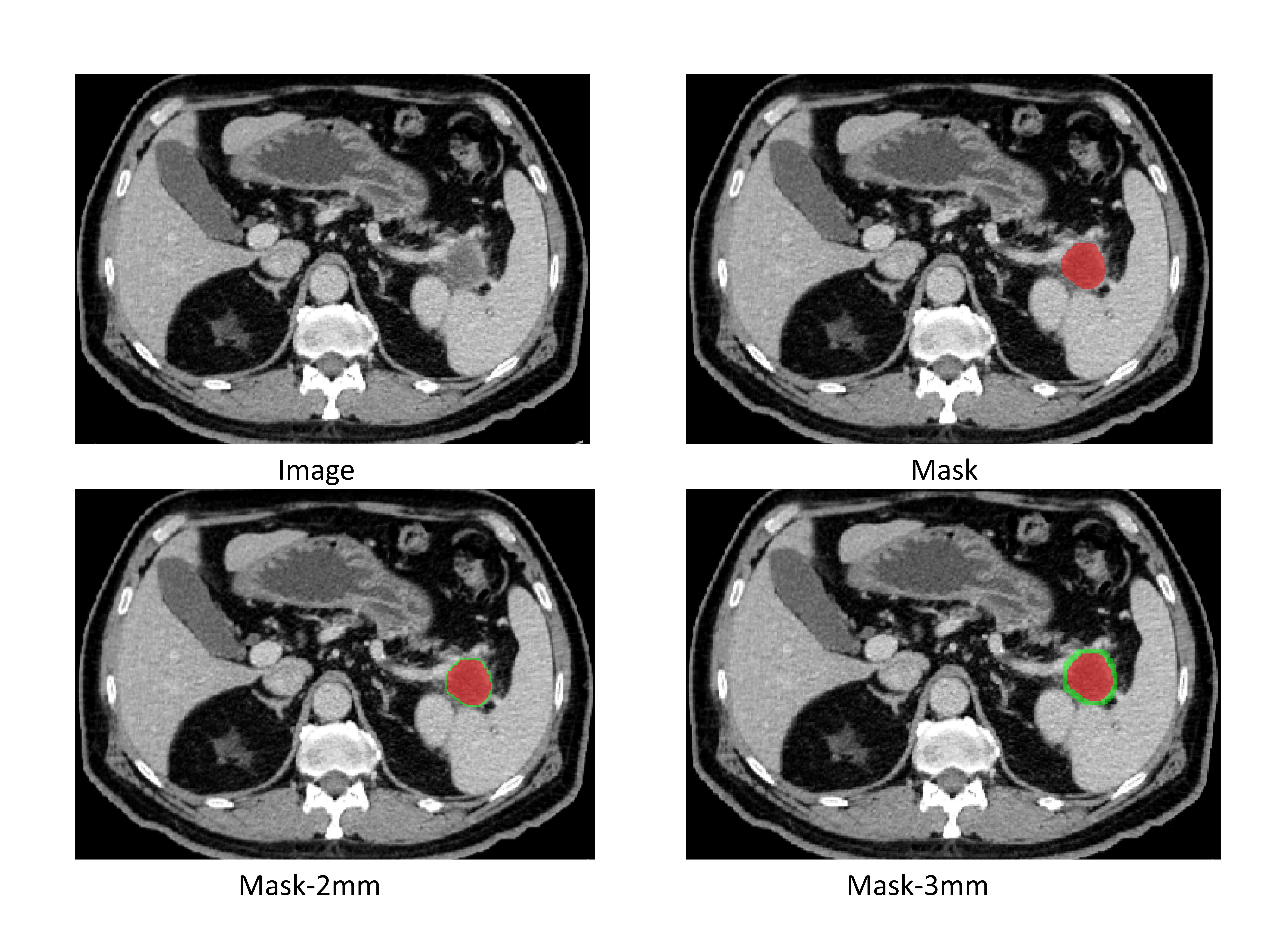

Supplement: Supplementary Figure 2 — Schematic illustration of the region of interest (ROI) segmentation and peritumoral margin dilation. [file Image2.tif]
